# Outcomes Following the Use of Extracellular Matrix Cartilage Allograft for the Management of Osteochondral Lesions of the Talus: A Systematic Review

**DOI:** 10.7759/cureus.62044

**Published:** 2024-06-10

**Authors:** James Butler, Hayden Hartman, Ravneet Dhilllon, Taylor Wingo, Luilly Vargas, Wendell W Cole, Samuel R Montgomery, Alan P Samsonov, Gino M Kerkhoffs, John G Kennedy

**Affiliations:** 1 Orthopedic Surgery, New York University (NYU) Langone Health, New York, USA; 2 Orthopedic Surgery, Lincoln Memorial University DeBusk College of Osteopathic Medicine, Knoxville, USA; 3 Orthopedic Surgery, Royal College of Surgeons in Ireland, Dublin, IRL; 4 Orthopedics, New York University (NYU) Langone Health, New York, USA; 5 Orthopedic Surgery, Amsterdam University Medical Center (UMC), Amsterdam, NLD

**Keywords:** osteochondral lesion, autologous osteochondral transplantation, ortho-biologics, cartilage, extracellular matrix cartilage allograft

## Abstract

Extracellular matrix cartilage allograft (EMCA) is a novel biological strategy utilized to augment the repair of osteochondral lesions of the talus (OLTs). However, there is no consensus on the precise role and outcomes following its use in the treatment of OLTs. The purpose of this systematic review was to evaluate the clinical and radiological outcomes following the use of EMCA for the treatment of OLT. During July 2023, the PubMed, Embase, and Cochrane Library databases were systematically reviewed to identify clinical studies examining outcomes following EMCA for the management of OLTs. In total, 162 patients (162 ankles) across five studies received EMCA as part of their surgical procedure at a weighted mean follow-up time of 23.8±4.2 months. Across all five studies, there were improvements in subjective clinical outcomes following the use of EMCA, regardless of the clinical scoring tool utilized. Two studies demonstrated superior postoperative magnetic resonance observation of cartilage repair tissue (MOCART) scores in the EMCA cohort compared to the bone marrow stimulation (BMS) cohort alone. In the EMCA-BMS cohort, there were seven complications (9%) and three failures (4.1%). In the autologous osteochondral transplantation (AOT) cohort, there were 10 complications (38.5%), zero failures, and six secondary surgical procedures (23.1%). In the EMCA alone cohort, there were zero complications and three failures (4.3%), all of which underwent an unspecified revision procedure. This current systematic review demonstrated improvements in both clinical and radiological outcomes following the use of EMCA for the treatment of OLTs. Further prospective comparative studies with longer follow-up times are warranted to determine the precise role of EMCA in the management of OLT.

## Introduction and background

Osteochondral lesions of the talus (OLTs) are one of the most common intra-articular pathologies of the ankle joint involving injury to the articular cartilage and/or underlying subchondral bone [1]. Diagnosis is often delayed due to a low index of suspicion together with poor sensitivity of plain film radiographs for detecting OLTs [2]. Treatment strategies are predicated on a multitude of factors, the most important of which is lesion size [3,4]. Smaller lesions (<100 mm^2^) are treated with reparative procedures including arthroscopic debridement, curettage, and bone marrow stimulation [3]. Larger lesions (>100 mm^2^) typically warrant more extensive replacement procedures including autologous osteochondral transplantation [5] and osteochondral allograft transplantation [6].

The native articular cartilage lining the talar dome lacks regenerative capacity, primarily due to its avascular nature [4]. In light of the poor regenerative capacity of the native cartilage, novel biological modalities have been developed to augment the biological milieu to provide improved cartilage repair, including platelet-rich plasma, concentrated bone marrow aspirate (cBMA), and scaffolds including extracellular matrix cartilage allograft (EMCA) [4]. EMCA is a pre-packaged dehydrated micronized allogenic cartilage scaffold containing predominantly type II collagen, hyaline cartilaginous growth factors, and proteoglycans that are theorized to promote chondrogenesis [7]. The current basic science literature has demonstrated that EMCA may be a potent biological adjunct in the treatment of osteochondral lesions [8]. Fortier et al. reported superior International Cartilage Repair Society repair scores and favorable T2-mapping MRI relaxation times following the treatment of osteochondral lesions of the knee with bone marrow stimulation (BMS) via microfracture augmented with EMCA compared to microfracture alone [8]. The utilization of EMCA in the management of OLTs has been investigated [7,9-12], but there appears to be no consensus regarding the outcomes nor optimal indications for the use of this acellular scaffold.

The purpose of this systematic review was to evaluate the clinical and radiological outcomes following the use of EMCA for the treatment of OLTs. In addition, the level of evidence (LOE) and quality of evidence (QOE) were assessed.

## Review

Search strategy

In July 2023, a systematic review of the MEDLINE, Embase, and Cochrane Library databases was conducted based on the Preferred Reporting Items for Systematic Reviews and Meta-Analyses (PRISMA) guidelines [13]. The search terms used were as follows: (biocartilage or (extracellular and matrix and cartilage and allograft) or (micronized and cartilage)) and (osteochondral or chondral) and (talus or talar or ankle). The inclusion and criteria are displayed in Table 1. After retrieving the data, two independent reviewers (J.J.B. and H.H., each with two years of experience as orthopedic researchers) screened the titles and abstracts for potentially eligible paper studies, which received full-text reviews. A senior author (J.G.K., with 20 years of experience as a foot and ankle orthopedic surgery attending) was consulted to help reach a final decision if a consensus could not be reached.

**Table 1:** Inclusion and exclusion criteria

Inclusion criteria	Exclusion criteria
At least one year follow-up	Less than one year follow-up
Human participants	Cadaver or animal studies
Minimum five patients per cohort	Less than five patients per cohort
Outcomes following the use of extracellular matrix cartilage allograft for the treatment of osteochondral lesions of the talus	Systematic reviews or case reports
Written in English	Written in foreign language

Assessment of LOE and methodological quality

The LOE of the included studies was graded in accordance with the Journal of Bone and Joint Surgery [14]. The methodological quality of clinical evidence (QOE) and risk of bias for non-randomized studies were assessed using the Risk of Bias in Non-randomised Studies of Interventions (ROBINS-I) tool by two independent reviewers [15]. A senior author was consulted if any discrepancies arose. ROBINS-I evaluates seven domains which may introduce bias into a particular study, including potential confounders, participant selection process, classification of interventions, deviations from intended interventions, missing data, measurement of outcomes, and selection of reported results. Based on these domains, an overall assessment of bias in the study is determined. The risk of bias can be evaluated as "low," "moderate," "serious," or "critical," with an option of "no information."

Data extraction and evaluation

Two independent reviewers extracted and assessed the data from the selected studies. Data on patient demographics, lesion characteristics, and surgical characteristics were collected. Subjective clinical outcomes, radiographic outcomes, complication rates, failure rates, and secondary surgical procedure rates were evaluated.

Statistical analysis

SAS software version 9.3 (SAS Institute) was used to perform all statistical analyses. Descriptive statistics were calculated for continuous and categorical variables. Continuous variables were reported as weighted means and estimated standard deviations. Categorical variables were reported as frequencies with percentages. A value of p<0.05 was considered statistically significant. 

Results

Search and Literature Selection

**Figure 1:**
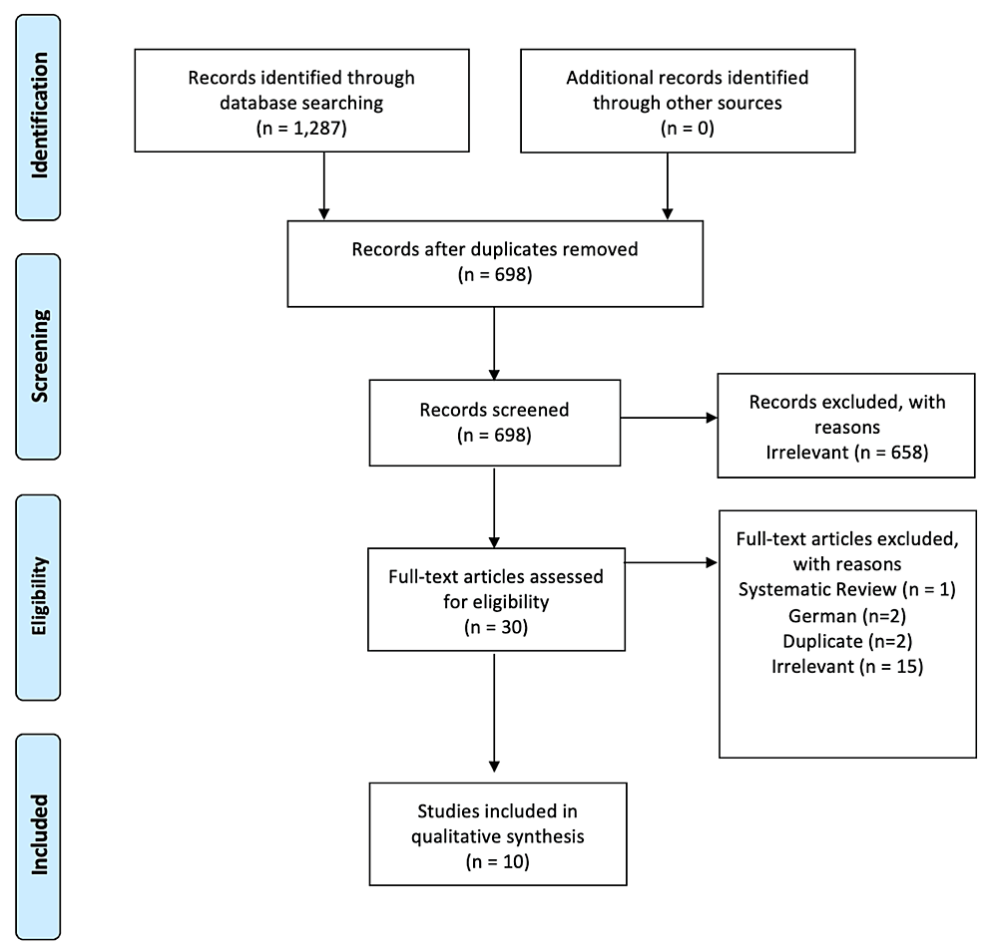
PRISMA flow diagram PRISMA: Preferred Reporting Items for Systematic Reviews and Meta-Analyses

The literature search revealed 43 studies, from which five studies were included in this review (Figure 1).

Study Characteristics and Patient Demographics

**Figure 2:**
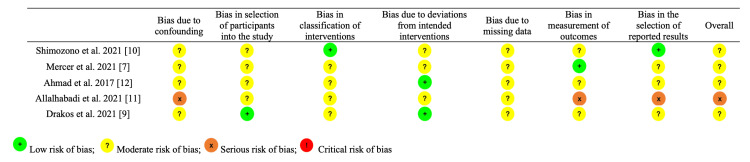
ROBINS-I ROBINS-I: Risk of Bias in Non-randomised Studies of Interventions

Study characteristics and patient demographic data are listed in Table 2. Four studies [7,9,10,12] were LOE III, and one study [11] was LOE IV. All five studies were of moderate risk of bias (Figure 2).

**Table 2:** Study characteristics and patient demographic data LOE: level of evidence; n: number; mo: months; y: years; yo: years old; M: male; F: female; R: right; L: left; BMI: body mass index; kg/m^2^: kilogram per meter squared

Author	LOE	Patients (n)	Ankles (n)	Follow-up (mo)	Age (y)	Sex (M/F)	R/L	BMI (kg/m^2^)	Smoking history
BMS augmented with EMCA									
Shimozono et al. 2021 [10]	3	24	24	20	47.8	11/13	13/11	-	-
Ahmad et al. 2017 [12]	3	30	30	20.2	40.7	17/13	12/18	-	-
Allahabadi et al. 2021 (EMCA+cBMA) [11]	3	14	14	25	32.9	4/2	-	27.7	0
Allahabadi et al. 2021 (EMCA+PRP) [11]	3	6	6	26	35.9	6/8	-	27.6	1
Arthroscopic debridement augmented with EMCA									
Drakos et al. 2021 [9]	3	62	62	23.5	36	-	-	27.33	-
AOT augmented with EMCA									
Mercer et al. 2021 [7]	3	26	26	31.3	36.7	10/16	14/12	-	0

In total, 162 patients (162 ankles) received EMCA as part of their treatment for OLT. The weighted mean postoperative follow-up time was 23.8±4.2 months (range, 20-31.2), and the weighted mean age was 38.4±5.3 years (range, 32.9-47.8). Forty-eight percent were males and 39 left ankles (48.8%). The mean BMI was 27.5±0.2 kg/m^2^ (range, 27.3-27.7). Seventeen patients (34.0%) had prior BMS, and one patient (0.7%) had a history of smoking.

Lesion and Surgical Characteristics

The pathological characteristics and surgical characteristics are listed in Table 3. The weighted mean area of the lesion was 78.0±42.6 mm^2^ (range, 31.3-136.6). Of the 162 OLTs, 111 (68.5%) were located medially, 45 (27.8%) laterally, and six (3.7%) centrally. There were 37 shoulder lesions (74.0%) in two studies [7,10] and 22 cystic lesions (44.0%).

**Table 3:** Pathological characteristics and surgical characteristics n: number; mo: months; BMS: bone marrow stimulation; EMCA: extracellular matrix cartilage allograft; AOT: autologous osteochondral transplantation; CBMA: concentrated bone marrow aspirate; MCM: micronized cartilage matrix; BMAC: bone marrow aspirate concentrate; PRP: platelet-rich plasma

Author	Patients (n)	Ankle (n)	Mean lesion area (mm^2^)	Lesion location			Shoulder lesions	Cystic lesions	History of prior microfracture	Technique
				Medial	Lateral	Central				
BMS augmented with EMCA										
Shimozono et al. 2021 [10]	24	24	31.3	13	9	2	16	7	8	BMS+EMCA
Ahmad et al. 2017 [12]	30	30	110	19	8	3	-	-	-	Arthroscopic excision+microfracture+ECM
Allahabadi et al. 2021 (EMCA+cBMA) [11]	14	14	106.5	14	0	0	-	-	-	Microfracture+MCM+BMAC
Allahabadi et al. 2021 (EMCA+PRP) [11]	6	6	45.9	6	0	0	-	-	-	Microfracture+MCM with PRP
Arthroscopic debridement augmented with EMCA										
Drakos et al. 2021 [9]	62	62	52.6	34	27	1	-	-	-	ECM-BMAC
AOT augmented with EMCA										
Mercer et al. 2021 [7]	26	26	136.6	25	1	0	21	15	-	AOT+CBMA/EMCA single graft=19; double graft=7

EMCA was utilized as a biological augment during arthroscopic BMS via microfracture in three studies [10-12], utilized as a biological augment during AOT in one study [7], and utilized as a biological augment during anterior ankle arthroscopic debridement in one study [9]. cBMA was utilized concomitantly in four studies [7, 9-11].

BMS Augmented with EMCA

Across three studies, there were 74 patients (74 ankles) who underwent BMS via microfracture augmented with EMCA for the treatment of OLT with a weighted mean follow-up time of 28.4±2.7 months (range, 20-26) [10-12]. The weighted mean lesion size was 116.4±40.7 mm^2^ (range, 31.3-110.0 mm^2^). One study reported 16 shoulder lesions (66.7%) and seven cystic lesions (29.2%) [10].

The clinical and radiographic outcomes are listed in Table 4. The Visual Analogue Scale (VAS) was utilized in two studies which improved from a preoperative weighted mean VAS of 7.3±1.6 (range, 5-8.1) to a postoperative weighted mean of 3.3±2.3 (range, 1.7-4.2). The Foot and Ankle Ability Measure (FAAM) was utilized in two studies which improved from a preoperative mean of 51.4 to a postoperative weighted mean of 90.4±4.4 (range, 89.3-97.7). The Foot and Ankle Outcome Score (FAOS) was utilized in one study which improved from a preoperative weighted mean of 49.9±2.0 (range, 47.8-50.7) to a postoperative weighted mean of 69.4±0.38 (range, 69.32-69.86) [10].

**Table 4:** Clinical outcomes, complications, failures, and secondary surgical procedures n: number; mo: months; FAOS: Foot and Ankle Outcome Score; VAS: Visual Analogue Scale; EMCA: extracellular matrix cartilage allograft; PRP: platelet-rich plasma; cBMA: concentrated bone marrow aspirate; MOCART: magnetic resonance observation of cartilage repair tissue

Author	Patients (n)	Ankles (n)	Patient-reported outcome measurement			Postoperative MRI	Complications (n)	Failures (n)	Secondary procedures	
			-	Preoperative	Postoperative					
BMS augmented with EMCA										
Shimozono et al. 2021 [10]	24	24	FAOS	47.8	69.9	MOCART: 76.3	Hypertrophy of the allograft cartilage=1	2	AOT=2	
Ahmad et al. 2017 [12]	30	30	VAS	8.1	1.7	-	Persistence of a nonuniform chondral surface and subchondral edema at the location of the particulate cartilage placement=2. Ankle arthritic changes=1. DVT=1	1	AOT=1	
Allahabadi et al. 2021 (EMCA+cBMA) [11]	14	14	VAS	5	4.2	-		0	0	
Allahabadi et al. 2021 (EMCA+PRP) [11]	6	6	VAS	6.5	6.3	-	Postoperative paresthesia=2	0	0	
Arthroscopic debridement augmented with EMCA										
Drakos et al. 2021 [9]	62	62	FAOS	50.68	69.3	MOCART: 73	0	3	"Revision procedure"=3	
AOT augmented with EMCA										
Mercer et al. 2021 [7]	26	26	FAOS	50.3	77.8	MOCART: 78.9	Knee donor site pain=2 (%). Painful hardware=3. Anterior ankle impingement=3	0	Anterior ankle impingement debridement=3. Hardware removal=3	

Postoperative radiographic data was obtained in one study [10]. The mean postoperative magnetic resonance observation of cartilage repair tissue (MOCART) was 76.3±14.9. Complete infill of the defect was reported in 14 patients (87.5%), complete integration to the border zone was reported in 13 patients (81.3%), and an intact subchondral lamina was present in 12 patients (75.0%). 

Complications, failures, and secondary procedure data and rates are listed in Table 4. In total, there were seven complications (9.5%). Complications included hypertrophy of the allograft cartilage (1.4%), persistence of a nonuniform chondral surface and subchondral edema at the location of the cartilage placement (2.7%), ankle arthritic changes (1.4%), postoperative deep vein thrombosis (1.4%), and postoperative paresthesia (2.7%). Three failures (4.1%) were recorded, all requiring AOT procedure at a weighted mean time of 23.7±19.3 months from the index procedure.

Arthroscopic Debridement Augmented with EMCA

One study compared outcomes between EMCA-cBMA as a biological adjunct during anterior arthroscopy and debridement of OLT and BMS via microfracture alone [9]. In the EMCA-cBMA cohort, 62 patients were included at a mean follow-up time of 23.5 months. The mean lesion size was 52.6 mm^2^. Fifty-five percent of lesions were located medially, 43.3% of lesions were located laterally, and 1.7% of lesions were located centrally. The authors did not report the number of shoulder nor cystic lesions.

In the EMCA-cBMA cohort, the Patient-Reported Measurement Information System (PROMIS) scores improved from preoperatively to postoperatively in the following domains: physical function 42.0±7.1 to 50.9±8.1; pain interference 58.6±7.4 to 49.9±9.5; pain intensity 47.2±8.9 to 39.0±8.5; global physical health 46.2±7.6 to 53.3±8.3; global mental health 51.8±10.1 to 52.8±9.2; and depression 48.8±9.3 to 46.7±8.0. The total FAOS score improved from a mean preoperative score of 49.4±17.7 to a postoperative score of 67.7±23.1. There was no statistically significant difference in PROMIS scores nor FAOS scores between the two cohorts.

Postoperative MRI scans were obtained in both cohorts. The mean MOCART score in the EMCA-cBMA cohort was 73±11.5 and was 54.0±24.1 in the microfracture alone cohort (p=0.0015). Complete or hypertrophic infill of the defect was observed in 85% of the EMCA-cBMA cohort and 59% of the microfracture cohort. Complete integration to the border zone was observed in 70% of the EMCA-cBMA cohort and 34% of the microfracture cohort.

No complications were recorded. Three failures (4.8%) were reported in the EMCA-cBMA cohort, and 14 failures (20.9%) were reported in the microfracture cohort (p=0.007). All three patients in the EMCA-cBMA cohort underwent a revision procedure at a mean time of 11.1 months following the index procedure. The authors did not specify the precise revision procedure performed.

AOT

One study compared outcomes between EMCA-cBMA as a biological adjunct during AOT and AOT alone [7]. In the EMCA-AOT cohort, 26 patients were included at a mean follow-up time of 31.3 months. The mean lesion size was 136.6±23.3 mm^2^. Twenty-five lesions were located medially (96.2%), one lesion was located laterally (3.8%), and no lesions were located centrally. There were 24 uncontained lesions (92.3%) and 15 cystic lesions (57.7%). Nine patients (34.6%) had a prior BMS via microfracture procedure. There were 19 single graft procedures (73.1%) and seven double graft procedures (26.9%).

In the EMCA-AOT cohort, the total FAOS score improved from a mean preoperative score of 50.3 to a postoperative score of 77.8. There was no statistically significant difference in FAOS scores between the two cohorts.

Postoperative MRI scans were obtained in both cohorts. The mean MOCART score in the EMCA-AOT cohort was 85.4±9.7, and the mean MOCART score in the AOT alone cohort was 78.9±14.5 (p=0.118). Eight postoperative (44%) were recorded in the EMCA-AOT cohort. 

Six complications (23.1%) were recorded in the EMCA-AOT cohort, which included knee donor site pain in two patients, painful hardware in three patients (11.5%), and anterior ankle impingement in three patients (11.5%). No failures were recorded. Six secondary surgical procedures (23.1%) were carried out, including three anterior ankle impingement debridement and three painful hardware removals. There was no statistically significant difference in complication rates nor failure rates between the two cohorts.

Discussion

The most important finding of this current systematic review was that the use of EMCA in the treatment of OLTs resulted in improvement in clinical outcomes with satisfactory postoperative MRI findings at short-term follow-up. Overall, there was a low complication and failure rate in this cohort. EMCA appeared to be an effective adjunct in the setting of BMS, but provided little benefit as an adjunct in patients undergoing AOT. Of note, the included studies were heterogeneous in nature and demonstrated poor QOE which did limit the ability to conduct any meaningful cross-sectional analyses.

The most common primary procedure performed across this systematic review was BMS via microfracture in three studies. Microfracture involves the use of various surgical instruments such as a pick, awl, or drill to perforate the subchondral plate which stimulates the aggregation of mesenchymal stem cells (MSCs) at the defect site [16,17]. This leads to the formation of a fibrin clot and the generation of fibrocartilaginous reparative tissue. Historically, microfracture was indicated to treat OLTs up to 150 cm^2^ in area; however, Ramponi et al. suggested that microfracture may be more suited to lesions smaller than 107.2 mm^2^ [18]. Furthermore, the concept of utilization of microfracture for smaller lesions was reinforced at the International Congress on Cartilage Repair of the Ankle consensus meeting in 2018 which concluded that microfracture is not indicated in patients with an OLT of >100 mm^2^ in area or >10 mm in diameter [16]. Although microfracture appears to produce favorable outcomes in the short-term period, there are concerns regarding the degradation of the fibrocartilaginous repair tissue in the long term [18]. During the initial six weeks following microfracture, the repair tissue de-differentiates from type II collagen into type I collagen and has reduced expression of proteoglycans and tissue fibrillation, ultimately producing an inferior "hyaline-like" substance [19]. Lee et al. demonstrated that there was inadequate integration of the reparative tissue in 70% of their cohort following BMS on second-look arthroscopy at one-year follow-up [20]. In addition, at five-year follow-up, Becher et al. found that 64% of their cohort had incomplete integration of the repair tissue with the border zone together with fibrillations in 100% of their patients [21]. Furthermore, concerns have been raised regarding the integrity of the subchondral plate and underlying subchondral bone following microfracture. The subchondral plate functions as a structural scaffold that bears 30% of the compressive articular load while communicating with the articular cartilage via cross-talk to facilitate numerous signaling pathways [22]. A systematic review of pre-clinical animal studies by Seow et al. found that BMS produced irreparable histological changes and reduced density of the architecture of the deep subchondral bone, ultimately predisposing the repaired construct to failure [23]. These concerning in vivo findings have been corroborated in clinical studies as evidenced by inferior clinical outcome scores and reduced subchondral bone health scores on MRI at mid-term follow-up after BMS by Shimozono et al. [22].

In light of the disparate deficiencies associated with microfracture, EMCA has been studied as a potential biological adjunct to improve the quality and integration of the reparative tissue with the adjacent native cartilage. This current systematic review found that BMS augmented with EMCA led to satisfactory improvement in subjective clinical outcomes at short-term follow-up, regardless of the subjective clinical outcome scoring tool utilized. Shimozono et al. conducted a retrospective comparative study between patients who received EMCA and patients who did not receive EMCA during BMS and found no statistically significant difference in postoperative FAOS scores between the two cohorts, suggesting that EMCA may not confer any practical advantage with regard to symptomatology [10]. In addition, the authors evaluated postoperative MOCART scores between the two cohorts and found superior MOCART scores in the EMCA cohort (76.3) compared to the BMS alone cohort (66.3), but this was not statistically significant (p=0.176). However, further analysis demonstrated that 87.5% of the EMCA cohort had complete infill of the defect compared to 46.5% of the BMS alone cohort. This suggests that although BMS augmented with EMCA produces similar subjective clinical outcomes compared to BMS alone in the short term, it may lead to superior reparative cartilaginous tissue, potentially improving the longevity of the joint. However, the lack of second-look arthroscopic examinations with focal tissue biopsy and histological analysis limits the generation of any robust conclusions regarding the quality of the reparative tissue.

Drakos et al. conducted a retrospective comparative study to evaluate outcomes between 67 patients who underwent microfracture for OLT and 62 patients who underwent arthroscopic implantation EMCA for OLT [9]. The authors performed anterior ankle arthroscopic debridement of any identified scar tissue, osteophytes, and loose fragments, and if a significant bone defect was present, concomitant bone grafting was also performed. This was followed by transplantation of EMCA which was subsequently secured with fibrin glue. The authors reported superior FAOS scores and PROMIS physical function and pain interference scores at short-term follow-up. Postoperative MRIs were obtained in 34 patients in the microfracture cohort and 20 patients in the EMCA cohort, which demonstrated superior MOCART scores in the EMCA cohort (73±11.5) compared to the microfracture cohort (53.9±24.1). Incomplete filling of the defect was observed in only 15% of the EMCA cohort compared to 41% of the microfracture cohort. This study suggests that the use of EMCA for small chondral injuries may be superior to microfracture alone, possibly due to the preservation of the integrity of the subchondral plate in the EMCA alone cohort. However, the discrepancy in lesion size between the two cohorts, follow-up period between the two cohorts, loss to follow-up, and lack of second-look arthroscopic examinations significantly limits the ability to advocate for the superiority of EMCA alone over BMS.

Large OLTs (>100 mm^2^) warrant replacement procedures such as AOT [5] and allogenic osteochondral transplantation [6]. AOT involves the resection of the diseased cartilage from the talus which is subsequently replaced with either an autologous single or double draft harvested from the non-weight-bearing portion of the ipsilateral lateral femoral condyle [5]. One study included in this current review compared outcomes between patients who underwent AOT augmented with EMCA and cBMA and patients who underwent AOT augmented with only cBMA [7]. At short-term follow-up, there was no statistically significant difference in postoperative FAOS scores between the two groups. It was postulated that the addition of EMCA would provide a biological hyaline-like grout at the graft-host interface which would subsequently lead to improved cartilage defect infill and reduce the incidence of postoperative subchondral cyst formation. However, no statistically significant difference was observed in mean MOCART scores between the two cohorts (p=0.118), suggesting that EMCA may not be a useful adjunct in AOT procedures. In addition, no difference in the rate of postoperative subchondral cysts was found between the two cohorts (p=0.8571), indicating that postoperative cysts may not be related to the degree of cartilage infill with the bone-graft interface; thus, EMCA may not mitigate against the development of postoperative cysts.

Four studies in this review utilized cBMA in addition to the EMCA scaffold. cBMA involves harvesting bone marrow from the ipsilateral iliac crest followed by centrifugation to produce a highly concentrated product of growth factors, MSCs, and a powerful anti-inflammatory, interleukin receptor 1 antagonist [24]. Recent in vitro studies have demonstrated that phagocytic monocytes, which are highly concentrated in cBMA, engulf local MSCs and lead to the formation of a paracrine signaling apparatus known as a secretome [24,25]. This specialized macrophage exerts its chondroprotective effect on the native cartilage via numerous immunomodulatory processes including the downregulation of IL-1β gene expression. cBMA has been studied extensively in the setting of OLTs, demonstrating superior infill when used in conjunction with BMS [26] and lower rates of postoperative cyst formation when utilized during AOT [27]. The addition of cBMA to the EMCA provides a three-dimensional biological scaffold populated by chondral secretomes, thus potentially improving the integration of the reparative cartilage into the native recipient talar cartilage. Although cBMA has an overall chondroprotective effect on the articular cartilage, a major drawback of this biologic is its propensity to develop excessive scar tissue formation. The TGF-β isoform that promotes cicatrized fibrous tissue formation is present in abundance in cBMA [24]. Intra-articular injection of cBMA in its liquid form is dissipated in synovial fluid soon after injection, while cBMA bound to the EMCA scaffold is not absorbed as rapidly which may cause prolonged potentiation of its pro-fibrotic effect, thus contributing to anterior ankle scar tissue formation. Mercer et al. reported an 11.5% incidence of anterior ankle impingement warranting surgical debridement of the cicatrized tissue in their cohort; thus, surgeons should be aware of the potential for anterior ankle impingement following the use of EMCA and cBMA in the treatment of OLTs.

Study limitations

This systematic review has inherent limitations due to the nature of the data reported leading to inconsistency between studies and challenges in developing firm conclusions. The highly heterogeneous nature of the patients and surgical protocols, varied indications, and underreporting of data were major confounders. The included studies were all of moderate risk of bias; thus, the results of this study must be interpreted with caution. Though there was one prospective study included, the retrospective nature of the included studies in addition to their lack of randomized control groups weakens the QOE and strength of conclusions.

## Conclusions

This current systematic review demonstrated improvements in both clinical and radiological outcomes following the use of EMCA for the treatment of OLTs. EMCA appeared to be an effective adjunct to BMS, with excellent subjective clinical outcomes and satisfactory postoperative MOCART scores reported at short-term follow-up. Although satisfactory postoperative outcomes following the use of EMCA as an adjunct to AOT were found at short-term follow-up, no significant benefit in comparison to AOT alone was observed, thus calling into question its utility in the setting of AOT. Further high-quality, prospective head-to-head comparative studies with longer follow-up times are warranted to determine the precise role of EMCA in the management of OLT. This current systematic review emphasizes the efficacy of EMCA as an adjunct to EMCA, but highlights its shortcomings in the setting of AOT.

## References

[1] Bruns J, Habermann C, Werner M (2021). Osteochondral lesions of the talus: a review on talus osteochondral injuries, including osteochondritis dissecans. Cartilage.

[2] Butler JJ, Wingo T, Kennedy JG (2023). Presurgical and postsurgical MRI evaluation of osteochondral lesions of the foot and ankle: a primer. Foot Ankle Clin.

[3] Arshad Z, Aslam A, Iqbal AM, Bhatia M (2022). Should arthroscopic bone marrow stimulation be used in the management of secondary osteochondral lesions of the talus? A systematic review. Clin Orthop Relat Res.

[4] Azam MT, Butler JJ, Duenes ML, McAllister TW, Walls RC, Gianakos AL, Kennedy JG (2023). Advances in cartilage repair. Orthop Clin North Am.

[5] Flynn S, Ross KA, Hannon CP (2016). Autologous osteochondral transplantation for osteochondral lesions of the talus. Foot Ankle Int.

[6] Migliorini F, Maffulli N, Baroncini A, Eschweiler J, Knobe M, Tingart M, Schenker H (2022). Allograft versus autograft osteochondral transplant for chondral defects of the talus: systematic review and meta-analysis. Am J Sports Med.

[7] Mercer NP, Samsonov AP, Dankert JF, Kennedy JG (2022). Outcomes of autologous osteochondral transplantation with and without extracellular matrix cartilage allograft augmentation for osteochondral lesions of the talus. Am J Sports Med.

[8] Fortier LA, Chapman HS, Pownder SL, Roller BL, Cross JA, Cook JL, Cole BJ (2016). BioCartilage improves cartilage repair compared with microfracture alone in an equine model of full-thickness cartilage loss. Am J Sports Med.

[9] Drakos MC, Eble SK, Cabe TN (2021). Comparison of functional and radiographic outcomes of talar osteochondral lesions repaired with micronized allogenic cartilage extracellular matrix and bone marrow aspirate concentrate vs microfracture. Foot Ankle Int.

[10] Shimozono Y, Williamson ER, Mercer NP, Hurley ET, Huang H, Deyer TW, Kennedy JG (2021). Use of extracellular matrix cartilage allograft may improve infill of the defects in bone marrow stimulation for osteochondral lesions of the talus. Arthroscopy.

[11] Allahabadi S, Johnson B, Whitney M, Oji D, Chou L, Lau BC (2022). Short-term outcomes following dehydrated micronized allogenic cartilage versus isolated microfracture for treatment of medial talar osteochondral lesions. Foot Ankle Surg.

[12] Ahmad J, Maltenfort M (2017). Arthroscopic treatment of osteochondral lesions of the talus with allograft cartilage matrix. Foot Ankle Int.

[13] Liberati A, Altman DG, Tetzlaff J (2009). The PRISMA statement for reporting systematic reviews and meta-analyses of studies that evaluate healthcare interventions: explanation and elaboration. BMJ.

[14] Wright JG, Swiontkowski MF, Heckman JD (2003). Introducing levels of evidence to the journal. J Bone Joint Surg Am.

[15] Sterne JA, Hernán MA, Reeves BC (2016). ROBINS-I: a tool for assessing risk of bias in non-randomised studies of interventions. BMJ.

[16] Hannon CP, Bayer S, Murawski CD (2018). Debridement, curettage, and bone marrow stimulation: proceedings of the International Consensus Meeting on Cartilage Repair of the Ankle. Foot Ankle Int.

[17] Hayashi S, Nakasa T, Ishikawa M, Nakamae A, Miyaki S, Adachi N (2018). Histological evaluation of early-phase changes in the osteochondral unit after microfracture in a full-thickness cartilage defect rat model. Am J Sports Med.

[18] Ramponi L, Yasui Y, Murawski CD (2017). Lesion size is a predictor of clinical outcomes after bone marrow stimulation for osteochondral lesions of the talus: a systematic review. Am J Sports Med.

[19] Shapiro F, Koide S, Glimcher MJ (1993). Cell origin and differentiation in the repair of full-thickness defects of articular cartilage. J Bone Joint Surg Am.

[20] Lee KB, Bai LB, Yoon TR, Jung ST, Seon JK (2009). Second-look arthroscopic findings and clinical outcomes after microfracture for osteochondral lesions of the talus. Am J Sports Med.

[21] Becher C, Driessen A, Hess T, Longo UG, Maffulli N, Thermann H (2010). Microfracture for chondral defects of the talus: maintenance of early results at midterm follow-up. Knee Surg Sports Traumatol Arthrosc.

[22] Shimozono Y, Coale M, Yasui Y, O'Halloran A, Deyer TW, Kennedy JG (2018). Subchondral bone degradation after microfracture for osteochondral lesions of the talus: an MRI analysis. Am J Sports Med.

[23] Seow D, Yasui Y, Hutchinson ID, Hurley ET, Shimozono Y, Kennedy JG (2019). The subchondral bone is affected by bone marrow stimulation: a systematic review of preclinical animal studies. Cartilage.

[24] Cassano JM, Kennedy JG, Ross KA, Fraser EJ, Goodale MB, Fortier LA (2018). Bone marrow concentrate and platelet-rich plasma differ in cell distribution and interleukin 1 receptor antagonist protein concentration. Knee Surg Sports Traumatol Arthrosc.

[25] Fortier LA, Strauss EJ, Shepard DO, Becktell L, Kennedy JG (2019). Biological effects of bone marrow concentrate in knee pathologies. J Knee Surg.

[26] Hannon CP, Ross KA, Murawski CD (2016). Arthroscopic bone marrow stimulation and concentrated bone marrow aspirate for osteochondral lesions of the talus: a case-control study of functional and magnetic resonance observation of cartilage repair tissue outcomes. Arthroscopy.

[27] Shimozono Y, Yasui Y, Hurley ET, Paugh RA, Deyer TW, Kennedy JG (2019). Concentrated bone marrow aspirate may decrease postoperative cyst occurrence rate in autologous osteochondral transplantation for osteochondral lesions of the talus. Arthroscopy.

